# Another Champion in the Field

**Published:** 1869-08

**Authors:** 


### EDITORIAL DEPARTMENT.

*Another Champion in the Field.*—After a cessation of hostilities for some months, the war against the formation of a "Southern Dental Association" has been renewed by the appearance of a new champion for the opposition, one of the editors of the *Dental Register*, who, over the signature of "W." endeavors to conceal his vexation under the guise of humor. While he accuses us of feeling sore, he cannot forgive our omission of a foot-note he took the *liberty* to add to Dr. Morgan's article as published in the *Register*, and which we considered to be a reflection upon Dr. M's veracity.

We professed to give our readers Dr. Morgan's statement, and do not admit that we were under any obligation to add the comments "W." was pleased to make concerning it; the more so as we regard Dr. M's word to be as reliable as that of "W."

When one has so far succeeded in the accomplishment of a cherished object, that there is every reason for believing that success is but a question of time, there can certainly be no cause for the existence of any of the sore feeling so humorously depicted by "W." But we rather suspect that "W." with his usual foresight, has been for some time convinced that the formation of a "Southern Dental Association," was no longer a matter for doubt; hence the bitterness of the pill.

